# Molecular cytogenetic characterization of a novel wheat–*Psathyrostachys huashanica* Keng T3DS-5NsL•5NsS and T5DL-3DS•3DL dual translocation line with powdery mildew resistance

**DOI:** 10.1186/s12870-020-02366-8

**Published:** 2020-04-15

**Authors:** Jiachuang Li, Li Zhao, Xueni Cheng, Guihua Bai, Mao Li, Jun Wu, Qunhui Yang, Xinhong Chen, Zujun Yang, Jixin Zhao

**Affiliations:** 1 Shaanxi Key Laboratory of Plant Genetic Engineering Breeding, College of Agronomy, Northwest A&F University, Yangling, 712100 Shaanxi China; 2 College of Life Science, Northwest A&F University, Yangling, 712100 Shaanxi China; 3 USDA, Hard Winter Wheat Genetics Research Unit, 4008 Throckmorton Hall, Manhattan, KS 66506 USA; 4 School of Life Science and Technology, University of Electronic Science and Technology of China, Chengdu, 610054 Sichuan China

**Keywords:** In situ hybridization, Dual translocation line, *Psathyrostachys huashanica*, Single nucleotide polymorphism array, Wheat powdery mildew

## Abstract

**Background:**

*Psathyrostachys huashanica* Keng (2*n* = 2*x* = 14, NsNs) carries many outstanding agronomic traits, therefore is a valuable resource for wheat genetic improvement. Wheat–*P. huashanica* translocation lines are important intermediate materials for wheat breeding and studying the functions of alien chromosomes. However, powdery mildew resistance in these translocation lines has not been reported previously.

**Results:**

This study developed a novel wheat–*P. huashanica* translocation line TR77 by selecting a F_7_ progeny from the cross between heptaploid hybrid H8911 (2*n* = 7*x* = 49, AABBDDNs) and durum wheat line Trs-372. Chromosome karyotype of 2*n* = 42 = 21II was observed in both mitotic and meiotic stages of TR77. Genomic in situ hybridization analysis identified two translocated chromosomes that paired normally at meiosis stage in TR77. Molecular marker analysis showed that part of chromosome 5D was replaced by part of alien chromosome fragment 5Ns. It meant replacement made part 5DL and part 5NsL**·**5NsS existed in wheat background, and then translocation happened between these chromosomes and wheat 3D chromosome. Fluorescence in situ hybridization demonstrated that TR77 carries dual translocations: T3DS-5NsL**·**5NsS and T5DL-3DS**·**3DL. Analysis using a 15 K-wheat-SNP chip confirmed that SNP genotypes on the 5D chromosome of TR77 matched well with these of *P. huashanica,* but poorly with common wheat line 7182. The translocation was physically located between 202.3 and 213.1 Mb in 5D. TR77 showed longer spikes, more kernels per spike, and much better powdery mildew resistance than its wheat parents: common wheat line 7182 and durum wheat line Trs-372.

**Conclusions:**

TR77 is a novel stable wheat–*P. huashanica* T3DS-5NsL**·**5NsS and T5DL-3DS**·**3DL dual translocation line and showed significant improved spike traits and resistance to powdery mildew compared to its parents, thus, it can be an useful germplasm for breeding disease resistance and studying the genetic mechanism of dual translocations.

## Background

Wheat (*Triticum aestivum* L, 2*n* = 42, AABBDD) is one of the most widely cultivated crops throughout the world. Also, wheat yield can be influenced by heat, drought, insects and diseases, while relatively narrow genetic variation in wheat makes it even more difficulty to identify sources of resistance to combat these biotic and abiotic stresses [1, 2]. Wheat wild relatives, however, carry many beneficial genes that can be used to improve wheat resistance to these stresses as well as grain yield and quality [3]. For example, a wheat–*Agropyron* 6P chromosome disomic addition line has more kernels per spike than its wheat parent [4]; a wheat–*Aegilops* substitution line called PRH_5_ showed high iron and zinc content [5]; a wheat–*Haynaldia villosa* 4DL–4VS translocation line called NAU413 exhibited high resistance to wheat spindle streak mosaic virus [6]; and wheat–rye 1BL/1RS translocation lines have been widely used in wheat breeding to improve resistance to multiple diseases and abiotic stress [3, 7].

*Psathyrostachys huashanica* Keng (2*n* = 2*x* = 14, NsNs) is an endangered species of *Poaceae*, *Triticeae* found only in high mountains of China. As a wild relative of wheat, it owns many outstanding traits, such as high tolerance to abiotic stress, i.e. salinity, alkalinity, cold and drought and high resistance to normal wheat diseases, i.e. rust, take-all and powdery mildew [8–11]. Our laboratory made a wide cross between *P. huashanica* and common wheat line 7182 in the 1980s and obtained the F1 hybrid, H811 (2*n* = 28, ABDNs), using in vitro culture of young embryos. The colchicine treated H811 was crossed and backcrossed to 7182 to get heptaploid hybrid H8911 (2*n* = 49, AABBDDNs). Subsequently, H8911 was then backcrossed to 7182 or crossed to several other wheat cultivars, and generated a series of wheat–*P. huashanica* derived lines including wheat–*P. huashanica* 1Ns–7Ns disomic addition lines [12–17], 2Ns(2D) disomic substitution line [18], 5Ns(5D) disomic substitution line [19], and translocation lines [20, 21]. These wheat–*P. huashanica* derived lines have more desired agronomic traits than their wheat parents, demonstrating that *P. huashanica* is a useful wild relative for wheat improvement. However, all the known wheat–*P. huashanica* translocation lines are poorly characterized [20–23]. Although previous studies identified the transposition of wheat and *P. huashanica* chromosomes in some wheat–*P. huashanica* derived lines, stability of these alien chromosomes in wheat, their homeologous relationship to wheat chromosomes, and actual translocation sites in wheat chromosomes remain to be investigated. Therefore, determining chromosomal composition of stable wheat–*P. huashanica* translocation lines may facilitate applications of this type of resources in wheat breeding.

Disease resistance genes from wild relatives of wheat play important roles in control of powdery mildew. Among the 25 wheat closely related genera, 14 demonstrated immunity to powdery mildew, including *Secale* [24], *Aegilops* [25], *Thinopyrum* [26], and *Haynaldia villosa* [27]. *P. huashanica* is also immune to powdery mildew but wheat–*P. huashanica* derived lines with powdery mildew resistance have not been reported to date.

In this study, we developed a novel wheat–*P. huashanica* translocation line with resistance to powdery mildew from a F_7_ progeny of wheat–*P. huashanica* heptaploid line H8911 × durum wheat (*Triticum durum* L, 2*n* = 28, AABB) line Trs-372. The objectives of this study were to: (a) determine the pairing and inherent stability of the transposed alien chromosome segment using cytogenetic methods; (b) examine the chromosomal composition of the derived line using molecular markers and fluorescence in situ hybridization (FISH); (c) determine the physical locations of the translocation sites on the chromosomes using a wheat 15 K single nucleotide polymorphism (SNP) array; and (d) evaluate the agronomic traits of the new line to predict its potential use in wheat breeding.

## Results

### Mitotic cells of TR77

**Fig. 1:**
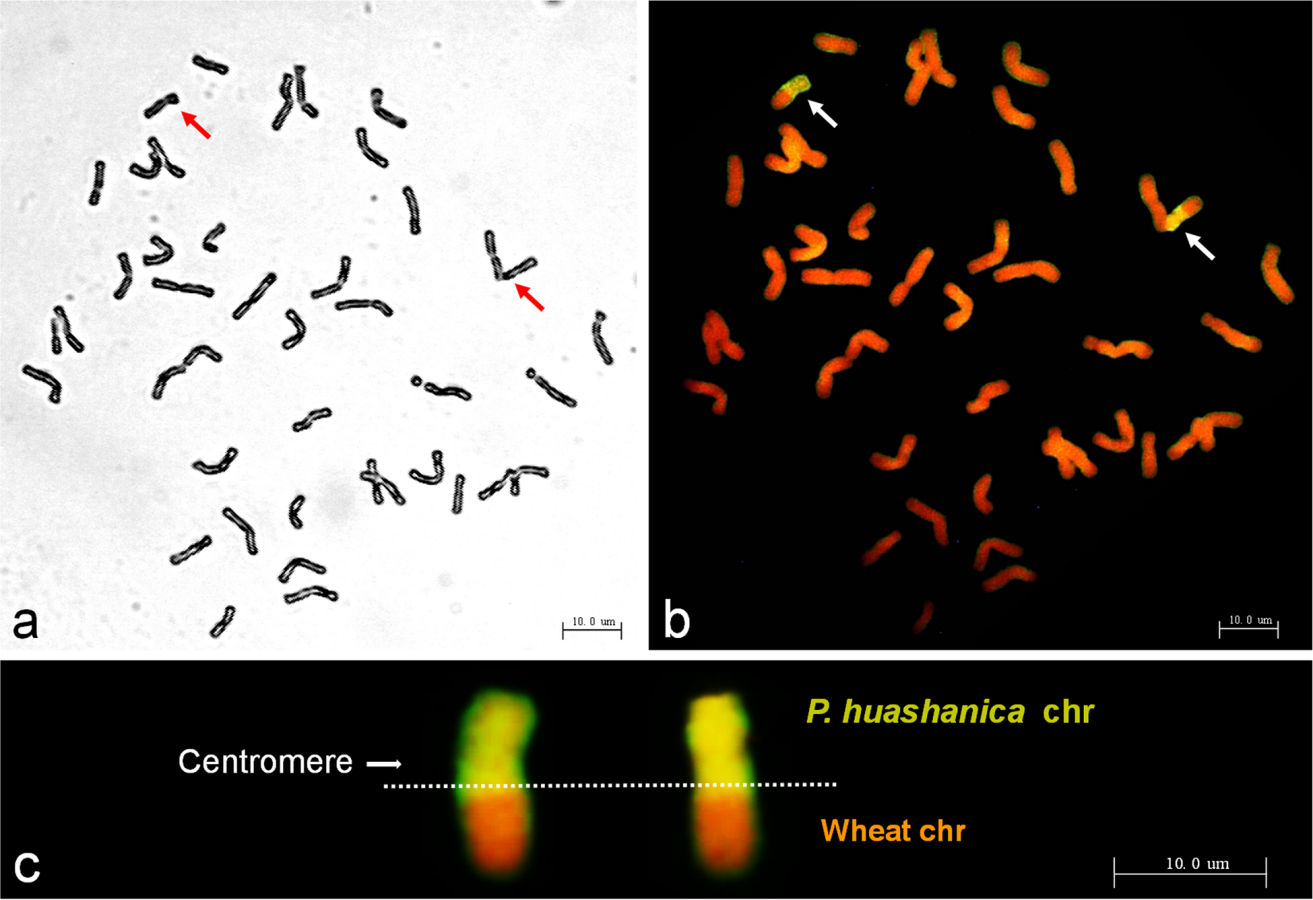
Cytological and genomic in situ hybridization (GISH) analyses of root tip cells from line TR77. **a** Mitotic metaphase, 2*n* = 42. **b** GISH analysis of TR77. GISH was conducted using the total DNA from *P. huashanica* as the probe and 7182 as the block. Two chromosomal segments with fluorescent hybridization signals (yellow-green) were detected as alien chromosomes in the mitotic metaphase. **c** Enlarged a pair of the translocated chromosomes. The alien chromosome segment consists of the short arm and a small portion of the long arm attached to the centromere. Chromosomes were counterstained with Propidium Iodide (red)

About 98% of metaphase mitotic root tip cells (RTCs) of TR77 had a chromosome number of 2*n* = 42 (Fig. 1a, Table 1) among 136 microscopically screened mitotic RTCs that were randomly selected from different plants. All the chromosomes were clearly separated from each other in metaphase. Genomic in situ hybridization (GISH) analysis using the whole genomic DNA from *P. huashanica* as the probe and wheat 7182 as the block on the same slide identified a pair of chromosomal segments with strong yellow-green hybridization signals (Fig. 1b). Each of the signals was emitted from nearly half of the wheat chromosome, and clearly covered the entire short arm and partial long arm of the chromosome that were connected through the centromere (Fig. 1c), indicating the wheat chromosomes were evidently transposed with *P. huashanica* chromosomal segments. Therefore, TR77 was a wheat–*P. huashanica* line with a large segment translocation.

**Table 1:** Chromosome numbers and pairing status in the meiotic and meiotic phases of TR77

Material	No. of counted cells	2*n*	Chromosome configuration					
			Univalent	Bivalent			Trivalent	Quadrivalent
				Rod	Ring	Total		
TR77	128	42	0.12 (0–2)	1.31 (1–3)	19.54 (19–21)	20.85 (20–21)	0	0

### Chromosomes in meiosis cells of TR77

**Fig. 2:**
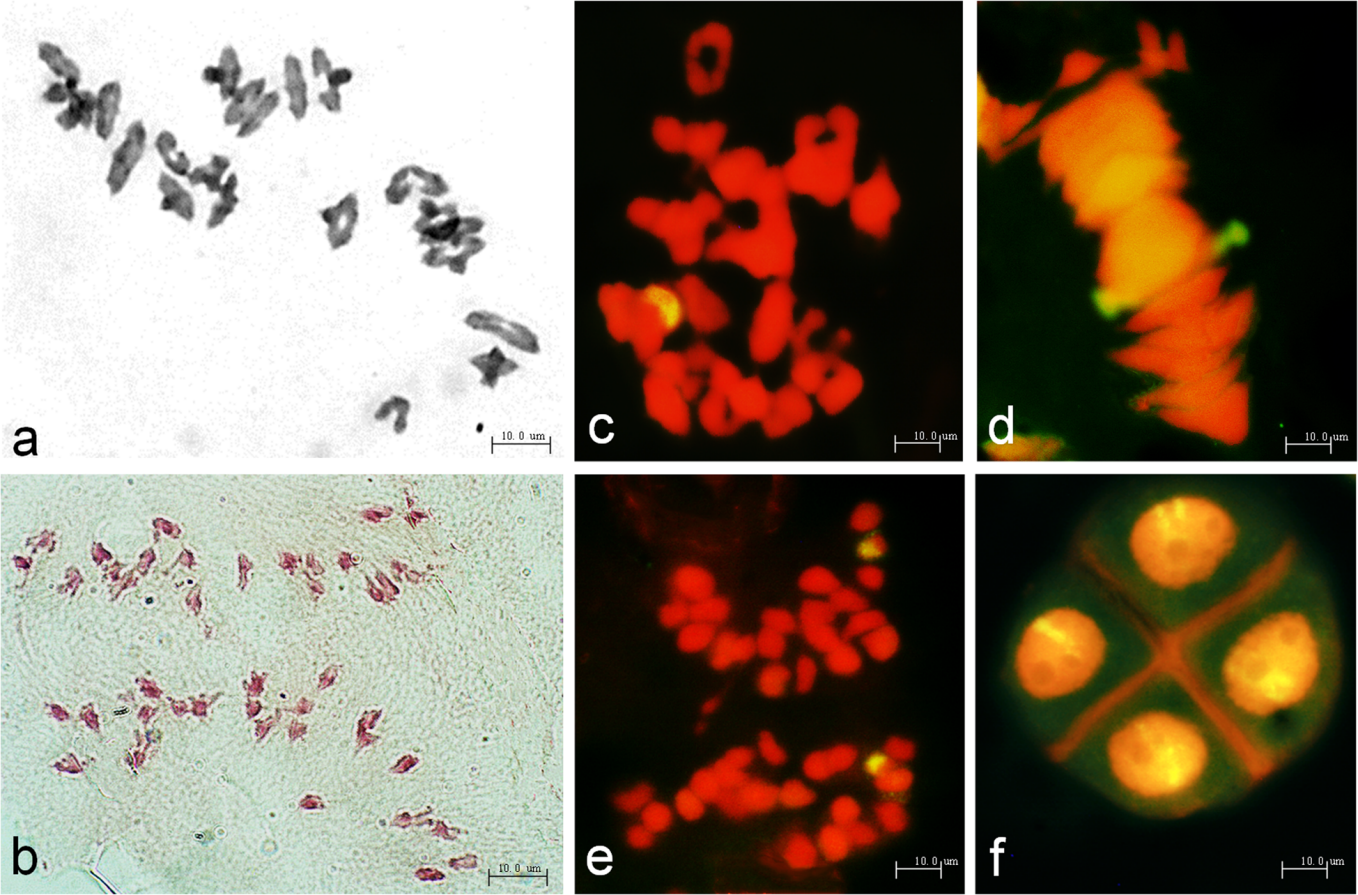
Cytological and genomic in situ hybridization analysis of pollen mother cells of line TR77. **a** Meiotic metaphase I, 2*n* = 21II. **b** Meiotic anaphase I, 2*n* = 21 + 21. **c** A tetrad with a semicircular fluorescent signal in meiosis prophase I. **d** Rod chromosome with partial yellow-green signal observed after the chromosomes arranged on the equatorial plate. **e** Two chromosomes with partial hybridization signals moved to the cell poles during meiotic anaphase I. **f** Each of the four progeny cells had a fluorescent signal in the meiosis telophase II period. *P. huashanica* chromosomes were labeled by the yellow-greenish fluorescent signals

Pollen mother cells (PMCs) in metaphase I of TR77 had a chromosome configuration of 2 *n* = 21II (Table 1, Fig. 2a). In meiosis anaphase I, trivalents or quadrivalents and lagging chromosomes were not observed (Fig. 2b). GISH analysis on PMCs in the important fission periods detected one tetrad with a semicircular fluorescent signal in meiosis prophase I (Fig. 2c), and then a rod chromosome with a partial yellow-green signal appeared when the chromosomes were arranged on the equatorial plate (Fig. 2d), separated into two chromosomes, and finally moved to the two poles of the cell in meiotic anaphase I (Fig. 2e). In meiosis telophase II, each of the four sperms carried a fluorescent segment (Fig. 2f). The pair of transposed *P. huashanica* chromosome fragments showed normal synapsis, pairing, segregation, and inheritance in the wheat background, therefore, they came from the same homoeologous groups, which confirmed that TR77 was a cytogenetically stable wheat–*P. huashanica* translocation line.

### Analysis of molecular markers in TR77

**Fig. 3:**
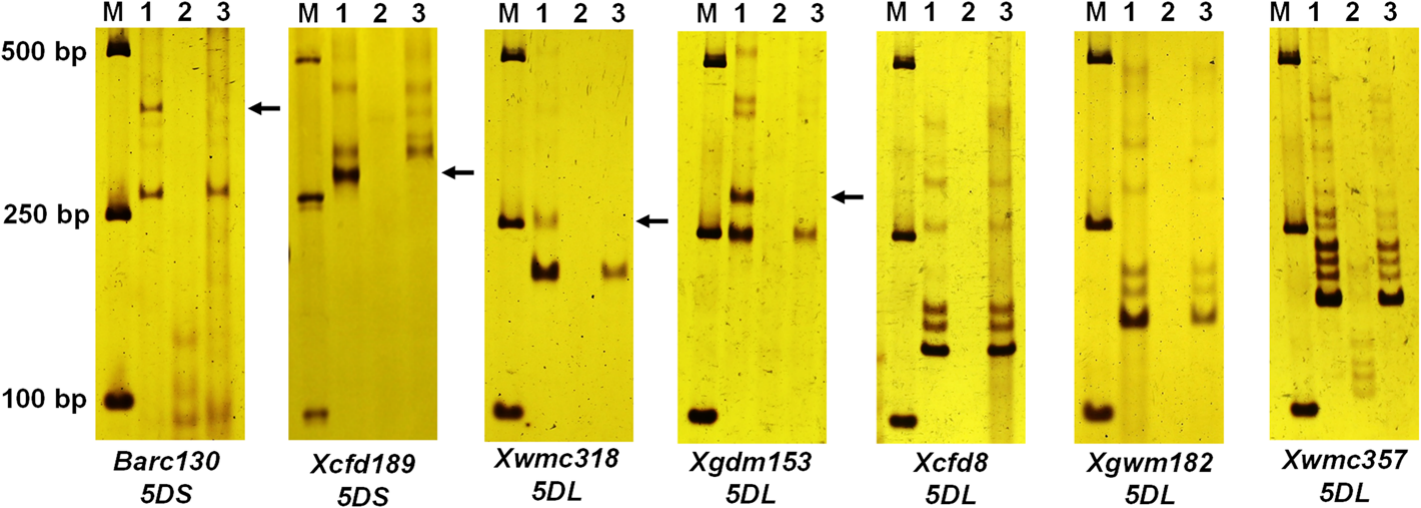
Genotype TR77 using simple sequence repeat (SSR) markers. The SSR markers *Xbarc130*, *Xcfd189*, *Xwmc318*, and *Xgdm153* did not amplify 5D genome-specific bands in TR77, but the markers *Xcfd8*, *Xgwm182* and *Xwmc357* amplified the same bands in TR77 as in the wheat parent 7182. Lane M: DL2000 marker; lane 1: line 7182; lane 2: line Trs-372; lane 3: line TR77*.* The missing D genome-specific bands were indicated by arrows

We screened 384 pairs of simple sequence repeat (SSR) primers distributed among the long and short arms of the wheat chromosomes to determine the homoeologous groups for the transposed wheat chromosomes in TR77 (Table S1, Figure S1). Except for 5D that have only a few markers amplified on 5DL of TR77, the markers on other wheat genomes amplified the same wheat chromosome specific bands between the wheat parent line 7182 and TR77, therefore TR77 might loss its whole short arm and part of the long arm of 5D chromosome. Further comparison of the SSR markers on the wheat 5D chromosome (Table 2, Fig. 3) between 7182 and TR77 found that the primers from 5DS and part of 5DL amplified wheat D genome-specific bands in the parent 7182, but not in TR77, confirming that the short arm and part of the long arm of the 5D chromosome were missing in the TR77, and the translocation position might be near the marker *Xcfd8* because the marker *Xgdm153* close to *Xcfd8* did not amplify in TR77.

**Table 2:** Wheat SSR and EST-STS markers used in this study to analyze the chromosomal composition of TR77

markers	locus	7182	Trs-372	TR77	***P***	Primer (5′-3′)	Tm (°C)
*Barc130*	5DS	**+**	**–**	**–**		**F:** CGGCTAGTAGTTGGAGTGTTGG **R:** ACCGCCTCTAGTTATTGCTCTC	50
*Xcfd189*	5DS	**+**	**–**	**–**		**F:** GCTAAAGCCACATAGGACGG **R:** GCACAAGATTTTGCAAGGCT	60
*Xwmc318*	5DL	**+**	**–**	**–**		**F:** CGTAAAATTACGGTGCATTGAT **R:** GTGGACTTTTGTGGTTTTTGAG	60
*Xgdm153*	5DL	**+**	**–**	**–**		**F:** TATAGGCAAATTAATTAAGACG **R:** ATCTTTATGTGAGTACACTGC	50
*Xcfd8*	5DL	**+**	**–**	**+**		**F:** ACCACCGTCATGTCACTGAG **R:** GTGAAGACGACAAGACGCAA	60
*Xgwm182*	5DL	**+**	**–**	**+**		**F:** TGATGTAGTGAGCCCATAGGC **R:** TTGCACACAGCCAAATAAGG	60
*Xwmc357*	5DL	+	**–**	+		**F:** TAGTGGGTGACCGGTCAAGA **R:** TGGACGGATTTGGTCATTTC	60
*BF146187*	5AS 5BS 5DS	–	**–**	+	+	**F:** CAAGGTGCAACAGTTCATGG **R:** GGTCACAGAAATATGCGGCT	60
*BE444644*	5AS 5BS 5DS	–	**–**	+	+	**F:** AAGCTTGCTGAGCTTTCTGG **R:** TTGAGGGATGTAGGGCAAAG	60
*CD452608*	5AS 5BS 5DS	–	**–**	+	+	**F:** TGATGTCTTGTCGTGGTCGT **R:** TTTTGGATGCAGCAAGACAG	60

*P* indicates *P. huashanica*; **+** means marker is present; − means marker is absent

**Fig. 4:**
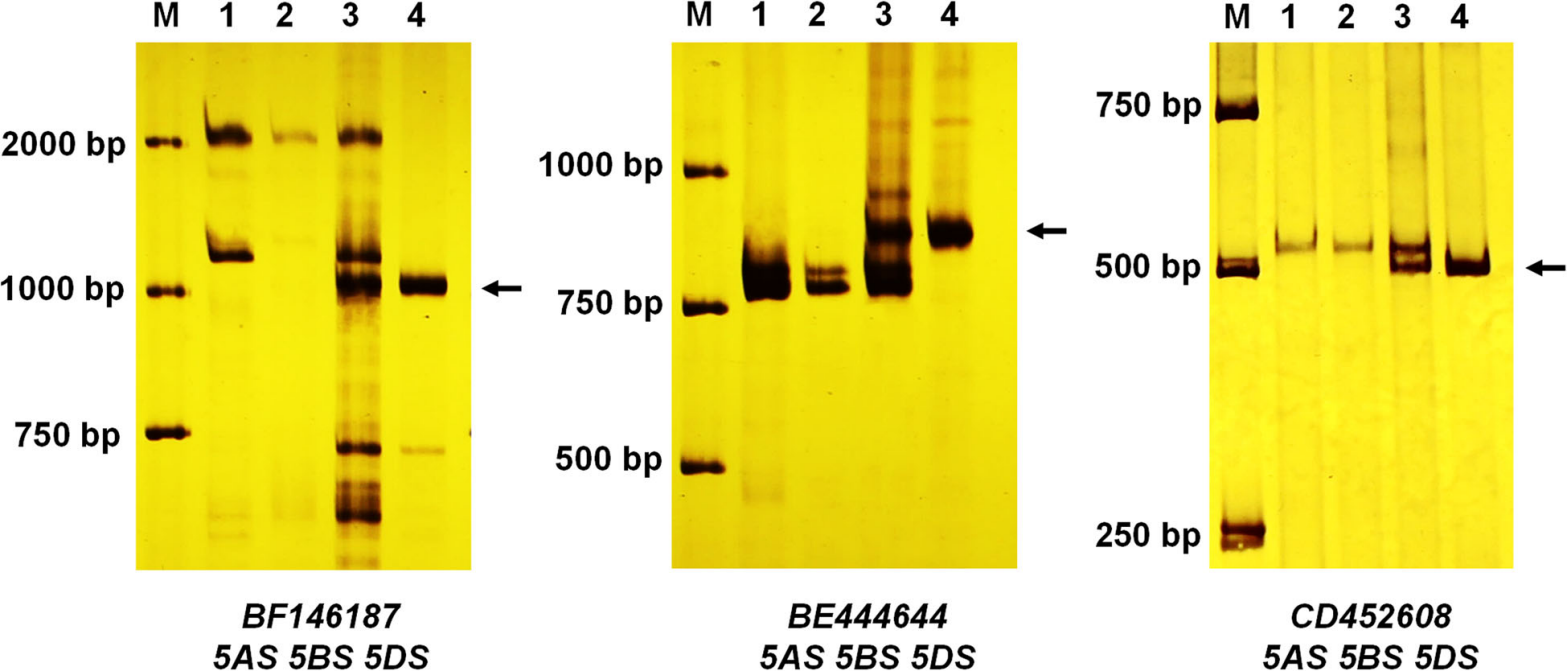
Expressed sequence tag-sequence tagged site (EST-STS) markers analysis of TR77 and its parents. Three pairs of EST-STS markers *BE444644*, *BF146187*, and *CD452608* corresponding homoeologous group 5 could amplify the Ns chromosome specific bands in *P. huashanica* and line TR77. Lane M: DL2000 marker; lane 1: line 7182; lane 2: line Trs-372; lane 3: line TR77; lane 4: *P. huashanica*. The diagnostic amplification products of Ns genome were indicated by arrows

Among the 78 pairs of expressed sequence tag-sequence tagged site (EST-STS) markers from seven wheat homoeologous groups, three pairs of primers, *BF146187*, *BE444644* and *CD452608,* from the wheat homoeologous group 5 (5AS, 5BS, and 5DS) (Table 2, Figure S1 and Fig. 4) amplified in *P. huashanica* and TR77, but not in wheat line 7182, suggesting that the three markers are Ns genome-specific and alien chromosome segment in line TR77 was from *P. huashanica* 5Ns chromosome.

### FISH analysis of TR77

**Fig. 5:**
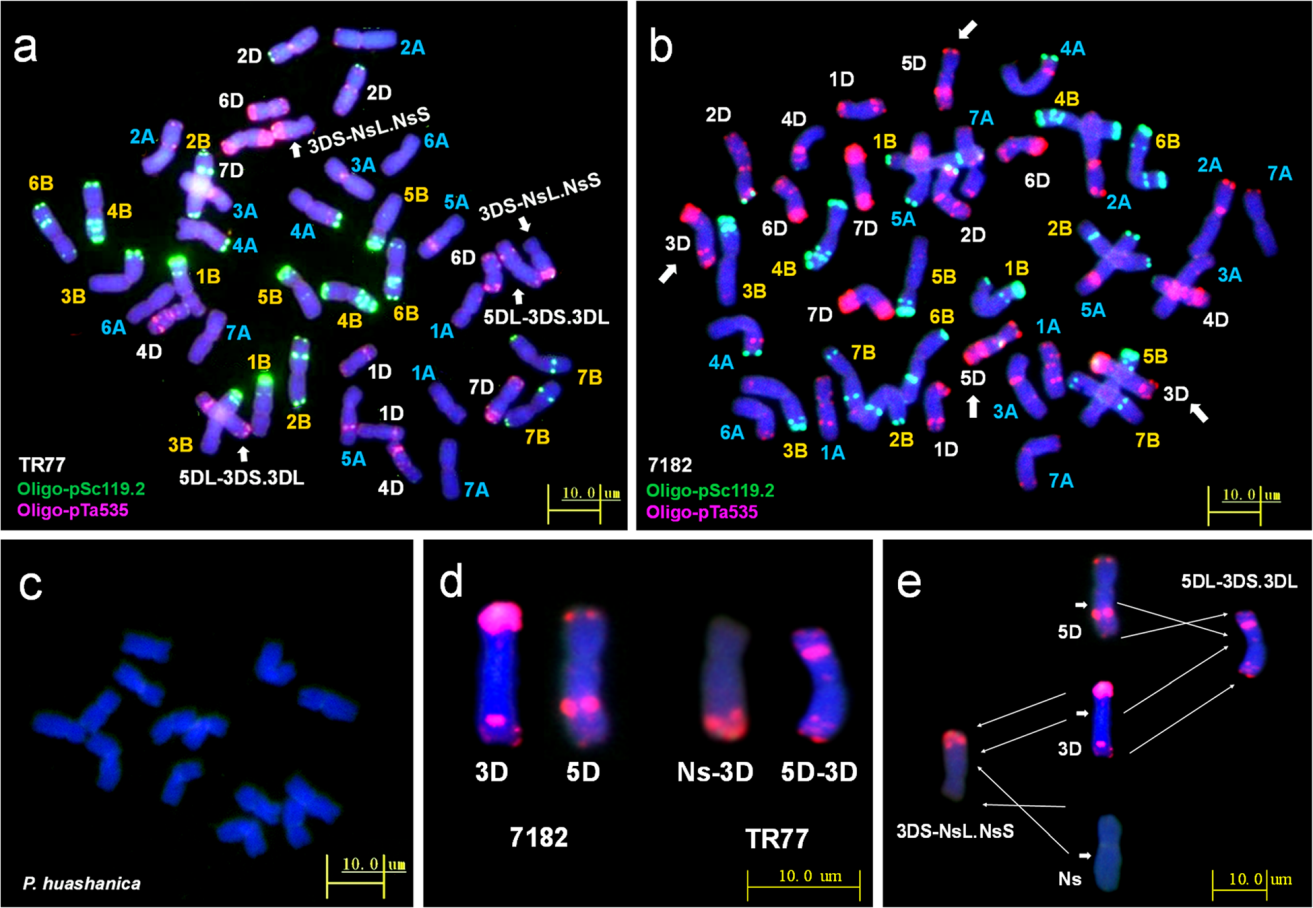
Fluorescence in situ hybridization (FISH) analysis of TR77. Oligo-primers pSc119.2 (green) and pTa535 (red) were used as probes for wheat chromosomes during the mitosis metaphase in the root tip cells. **a** line TR77. **b** common wheat 7182. **c***P. huashanica.* Wheat 3D and 5D chromosomes and chromosomes from two groups underwent double translocations to yield the 3DS-NsL.NsS and 5DL-3DS.3DL chromosomes as pointed by arrows. **d** Comparison of special chromosomes in common wheat 7182 and TR77. **e** Diagrammatic sketch of the two translocations, 3DS-NsL.NsS and 5DL-3DS.3DL, in TR77 to show breakage sites in each chromosome as pointed by arrows and chromosome rearrangement. Chromosomes were stained with 4′,6-diamidino-2-phenylindole (blue)

FISH analysis was performed to determine the transposed wheat chromosomes using repetitive oligonucleotide sequences pSc119.2 and pTa535 as probes. FISH results of line TR77, common wheat 7182 and *P. huashanica* could be seen in Fig. 5a, b and c, respectively. All the chromosomes of TR77 showed the same fluorescent karyotype as wheat line 7182 [28, 29], except for the chromosomes 3D and 5D (Fig. 5d) that showed translocations. One breakage site was observed between the long arm of 5D chromosome and short arm of 3D chromosomes of wheat (Fig. 5e), which was caused by loss of most portion wheat 5D chromosome and that remaining 5DL segment was combined with most portion of chromosome 3D (3DS**·**3DL) to become 5DL-3DS**·**3DL. The other breakage site was observed between the wheat 3DS chromosome and most portion of newly acquired *P. huashanica* chromosome 5Ns (NsL**·**NsS) segment (Fig. 5e) due to reciprocal translocations and recombination between remaining 3DS segment and the 5Ns segment (NsL**·**NsS) of *P. huashanica* to form a new translocation chromosome 3DS-NsL**·**NsS. Thus, TR77 was a translocation line with dual translocations.

### Comparison of fingerprints of TR77 and its parents using a SNP Array

**Fig. 6:**
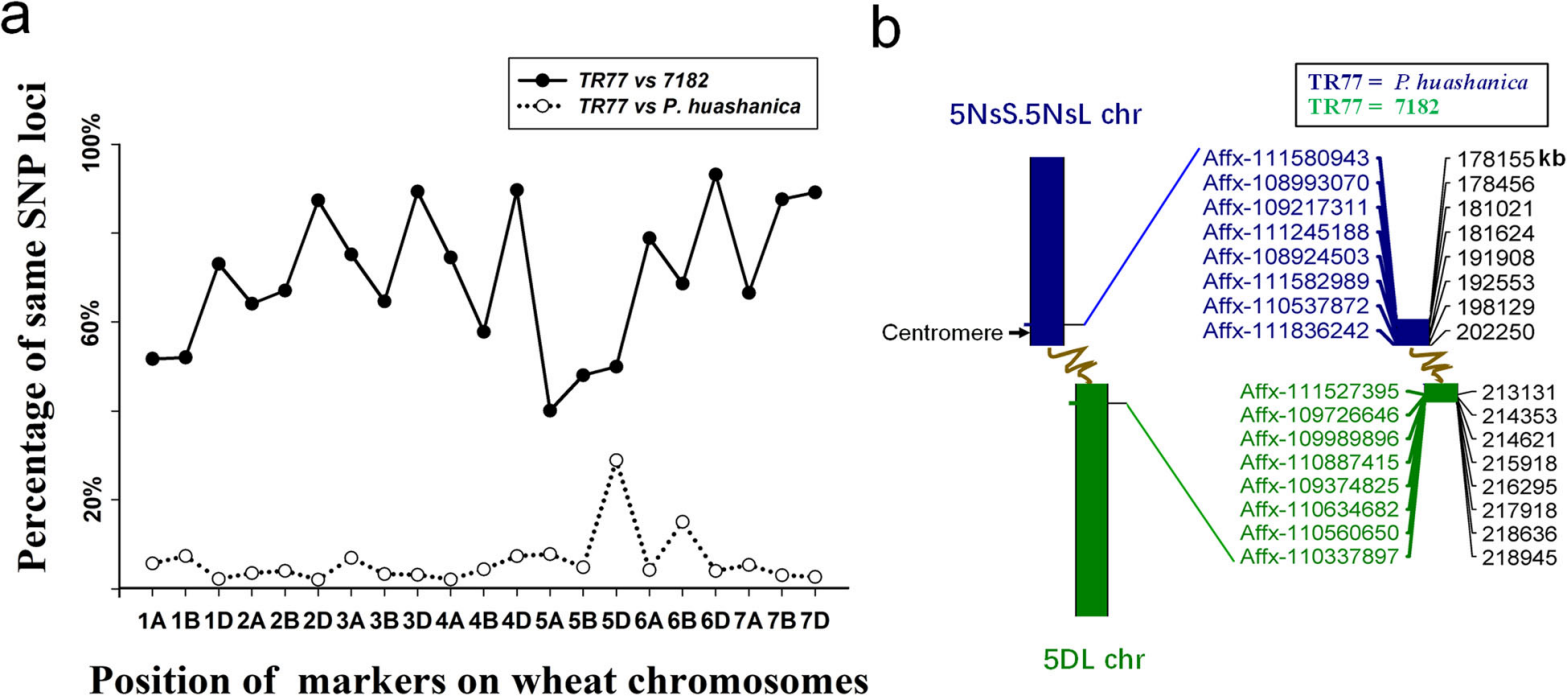
Chromosomal composition of TR77 analyzed using a wheat 15 K SNP array. **a** Difference in the 5D chromosomes according to the percentages of the same SNP loci. **b** The same genotype SNPs were arranged in the 5D chromosome between TR77 and its parents. An obvious boundary was determined between Affymetrix SNP ID Affx-111,836,242 and Affx-111,527,395, where the physical location was between 202.3 Mb and 213.1 Mb

To determine the chromosomal composition of TR77, wheat 15 K SNP arrays were employed to genotype TR77and its parents (Table S2). In general, significantly higher percentages of common SNP sequences on all 21 chromosomes were observed between TR77 and 7182 than between TR77 and *P. huashanica* except for the 5th group chromosomes that had the lowest percentages of the same SNP loci between TR77 and 7182 (Table 3, Fig. 6a) and the chromosome 5D that had the highest percentage of the same SNP loci between TR77 and the alien parent *P. huashanica*, confirming the translocation occurred between wheat and *P. huashanica* on the chromosome homeologous 5. After comparison of the corresponding SNP positions in chromosome 5D between TR77 and its parent 7182 (Fig. 6b), we found an obvious boundary between Affymetrix SNP ID Affx-111,836,242 and Affx-111,527,395 located between 202.3 Mb and 213.1 Mb of 5D chromosome and assumed that translocation occurred in this interval.

**Table 3:** Comparison of wheat 15 K SNP array data between TR77 and its parents

Chromosome	No. of markers	No. of valid markers in materials	No. of same markers (TR77 vs 7182)	Percentage of same markers (TR77 vs 7182)	No. of same markers (TR77 vs Trs-372)	Percentage of same markers (TR77 vs Trs-372)	No. of same markers (TR77 vs *P. huashanica*)	Percentage of same markers (TR77 vs *P. huashanica*)
1A	607	408	211	52%	304	75%	23	6%
1B	670	438	228	52%	229	52%	32	7%
1D	337	234	171	73%	–	–	5	2%
2A	907	607	389	64%	166	27%	21	3%
2B	736	431	289	67%	275	64%	17	4%
2D	597	404	353	87%	–	–	8	2%
3A	594	391	294	75%	149	38%	27	7%
3B	997	616	398	65%	432	70%	20	3%
3D	505	357	319	89%	–	–	11	3%
4A	766	545	406	74%	395	72%	11	2%
4B	589	348	201	58%	196	56%	15	4%
4D	248	165	148	90%	–	–	12	7%
5A	690	414	166	40%	203	49%	32	8%
5B	763	462	222	48%	286	62%	22	5%
5D	518	360	180	50%	–	–	104	29%
6A	463	265	209	79%	80	30%	11	4%
6B	768	446	306	69%	226	51%	67	15%
6D	404	279	260	93%	–	–	11	4%
7A	735	490	326	67%	320	65%	26	5%
7B	640	404	354	88%	228	56%	12	3%
7D	665	462	412	89%	–	–	12	3%
A genome	4762	3120	2001	64%	1617	52%	151	5%
B genome	5163	3145	1998	64%	1872	60%	185	6%
D genome	3274	2261	1843	82%	–	–	163	7%
Total	13,199	8526	5842	69%	3489	41%	499	6%

### Agronomic traits and resistance to powdery mildew in TR77

**Fig. 7:**
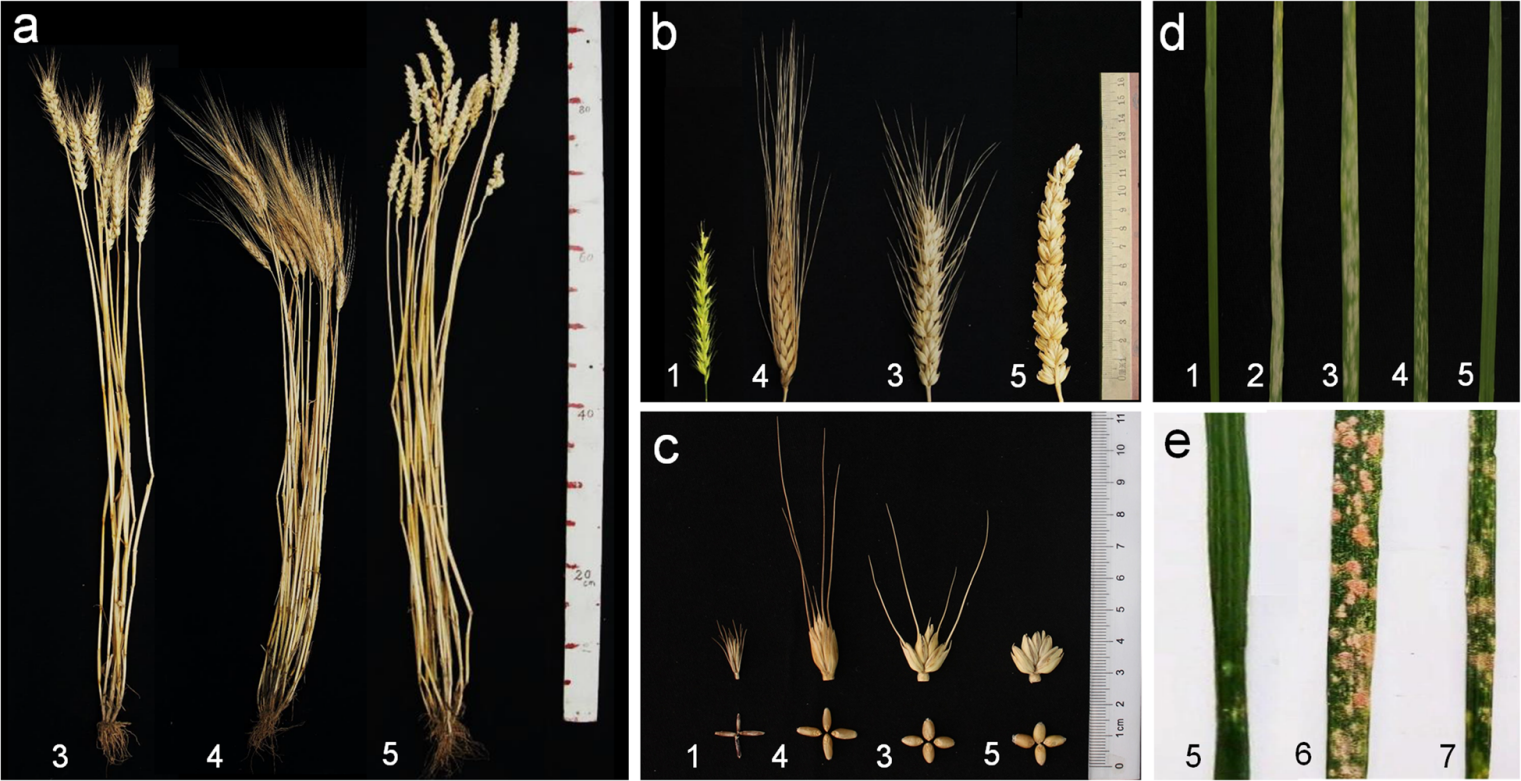
Agronomic traits and powdery mildew resistance in the translocation line TR77, its parents, and controls. **a** Plants. **b** Spikes. **c** Kernels. **d** Symptoms in response to inoculation with a mixture of *Bgt* race E09 races in the seedling stage. **e** Symptoms in response to powdery mildew after natural infection in the adult stage. The samples in the Figure are (1) *P. huashanica*; (2) Huixianhong; (3) 7182; (4) Trs-372; (5) TR77; (6) Mianyang 11 and (7) Chinese Spring

The agronomic traits were evaluated for TR77 and their parents (common wheat 7182, durum wheat Trs-372, *P. huashanica*) (Table 4 and Fig. 7). TR77 exhibited completely different spike traits from its wheat parent. TR77 had conical and awnless spikes, taller plant, longer spikes, and more kernels per spike than its parents (*P* < 0.01) between TR77 and its common wheat parent 7182 and Trs-372, indicating that addition of 5Ns chromosome of *P. huashanica* in TR77 increased spike length and spikelets number per spike, but also plant height.

**Table 4:** Agronomic traits of TR77, its parents, *P. huashanica,* common wheat 7182, and durum wheat Trs-372

Material	Plant height (cm)	Tiller number	Spike length (cm)	Spikelets per spike	Kernels per spike	Thousand kernel weight (g)	Awn type
*P. huashanica*	63.5 ± 2.2Dd	clump	8.26 ± 0.96BCbc	16 ± 2BCb	42 ± 6Cc	3.44 ± 0.11Cc	Short awn
7182	83.8 ± 1.4Bb	9 ± 2Ab	9.05 ± 0.57Bb	18 ± 3ABa	54 ± 4Bb	37.69 ± 0.74Aa	Long awn
Trs-372	76.3 ± 2.9Cc	10 ± 2Aab	7.91 ± 0.33Cc	14 ± 2Cb	43 ± 2Cc	35.52 ± 0.6Bb	Long awn
TR77	92.6 ± 2.3Aa	12 ± 3Aa	13.05 ± 0.77Aa	19 ± 3Aa	67 ± 5Aa	37.56 ± 0.69Aa	Awnless

Different uppercase and lowercase letters indicate significant differences at *p* < 0.01 and *p* < 0.05, respectively, between the translocation line TR77 and its parents

The response of TR77 to powdery mildew infection was evaluated in both a growth chamber and a field. Control varieties, TR77, and its parents were grown under the same conditions to ensure accurate results. In the seedling age, susceptible mildew infection types (IT) were observed in Huixianhong (IT = 4), Mianyang 11 (IT = 4), line 7182 (IT = 3) and Trs-372 (IT = 3), whereas *P. huashanica* (IT = 0) and TR77 (IT = 0;) were almost immune to powdery mildew (Fig. 7d). In the adult stage, the leaves of TR77 clearly had less spores than those of the control materials Mianyang 11 and Chinese Spring at the same positions on each plant (Fig. 7e).

## Discussion

Wheat is a self-pollinating plant, and thus it may not evolve rapidly enough to meet the needs of modern humans. Modern wheat breeders usually create desired genetic variations by making crosses between breeding lines within bread wheat or using mutagenesis, transgenic approach, etc. [30]. However, for mutagenesis, controlling the direction of mutations is very difficult due to the random nature of this process, the large and complex genome of allopolyploid wheat and quantitative nature of the most target traits, and transformation genotype specificity limit transgenic wheat breeding to a few traits in a few genotypes [31], making crossing breeding the most widely used strategy for creation of new variations in wheat breeding. However, long time interbreeding has narrowed the wheat genetic diversity, and caused loss of many useful genes for disease resistance, and plant adaptation and productivity. Wide crosses between wheat and its relatives can introduce alien genetic materials into common wheat to generate desired genetic variations and create new germplasm, which is important for the sustainable development of new wheat cultivars [32]. *P. huashanica* is a wheat relative species with many desirable traits that are valuable for wheat improvement. Many wheat lines carrying genetic materials from *P. huashanica* show outstanding agronomic traits. For example, a wheat–*P. huashanica* 1Ns disomic addition line exhibits increased storage of microelements in the seeds [10]. In addition, 2Ns, 3Ns, 4Ns, and 5Ns disomic addition lines and a 2Ns(2D) substitution line are resistant to stripe rust [13–16, 18]. A 6Ns disomic addition line and a small segment translocation line possess twin spikelets and more kernels per spike than its wheat parent [13, 21], and a 7Ns disomic addition line showed high resistance to leaf rust [12]. However, only a few wheat–*P. huashanica* translocation lines are available for breeding despite its importance. In the current study, we identified and characterized a novel wheat–*P. huashanica* translocation line that carries part of *P. huashanica* 5Ns chromosome and underwent dual reciprocal translocations in the wheat 3D and 5D chromosomes. This translocation line exhibited resistance to powdery mildew, an important wheat leave disease.

Whether alien chromosome segments can stably transmitted to their wheat offspring in a wheat background determines usefulness of the derived line in breeding [33]. In this study, the chromosomal compositions and behavior in the RTCs and PMCs of TR77 indicated 42 chromosomes with 21 bivalents in meiosis prophase I of TR77, with no lagging chromosomes during anaphase I. GISH analysis suggested that TR77 carries a pair of unique chromosomes that consist of chromosomal segments from both *P. huashanica* and wheat with the segment of *P. huashanica* from fifth homoeologous group (5Ns). The translocated 5Ns chromosome segment in TR77 paired normally in metaphase I and synchronized with the spliced wheat chromosome during the duplication and division phases, therefore, is stably inherited.

Genome specific DNA markers can be used to identify specific wheat chromosomes and determine homoeology of alien chromosome(s) with wheat. Du et al. [13] identified a wheat–*P. huashanica* 5Ns disomic addition line using EST-STS markers; Li et al. [19] identified a wheat–*P. huashanica* 5Ns(5D) disomic substitution line using SSR, EST-STS, sequence characterized amplified region (SCAR) markers. In this study, we used wheat genome-specific SSRs and *P. huashanica* genome specific EST-STSs to determine the chromosomal composition of TR77 and found that SSRs on 5DS and 5DL near the centromere amplified wheat genome-specific bands in parent 7182, but not in the translocation line TR77, whereas three EST-STSs on the fifth homoeologous group [13] amplified Ns genome-specific bands in both TR77 and *P. huashanica*. These results indicate that the short arm and partial long arm of wheat chromosome 5D close to the centromere were replaced by *P. huashanica* chromosome 5Ns. Many studies found that homoeologous chromosomes are remarkably conserved and any missing genetic content is usually complemented by corresponding homoeologous chromosomes [34–37]. In the H8911 × Trs-372 cross, because Trs-372 only carries A and B genomes, chromosome recombination mainly occurred between the D and Ns genomes, in particular, the reciprocal translocation between wheat 5D and *P. huashanica* 5Ns chromosomes that generated a new 5DL-5NsL**·**5NsS chromosome.

High-density SNP arrays can track sources of chromosomes from different species by comparing marker sequence identity between progeny and their donor parents. In the present study, comparison of a wheat 15 K SNP array data between TR77 and its parent 7182 confirmed that the D genome of TR77 was from 7182, whereas the A and B genomes were from chromosome recombination between wheat line 7182 and durum Trs-372. TR77 genome had extremely low similarity with the genome of *P. huashanica* except for the chromosome 5D of TR77 that had high percentage of SNP loci in common with *P. huashanica.* Also, 5D of TR77 had lower percentage of SNP loci in common with its wheat parent 7182 compared with the other chromosomes. These results indicated the translocation most likely occurred in wheat chromosome 5D, which was supported by the results from previous studies of DNA markers and GISH. To determine the translocation site, we arranged all SNP markers on the chromosome 5D based on their physical locations in Chinese Spring reference genome and identified an obvious physical boundary between 202.3 Mb and 213.1 Mb. The *P. huashanica* chromosomal segment was located before 202.3 Mb including the centromere region from 185.6 Mb to 188.7 Mb [38] and the common wheat (5DL) segment was located after 213.1 Mb of the chromosome. Therefore, translocation break position in TR77 is near the centromere of wheat 5D chromosome.

The FISH probes, Oligo-pSc119.2 and Oligo-pTa535, can accurately distinguish all 42 common wheat chromosomes based on the standard FISH karyotypes in mitotic cells [39, 40]. Many wheat-derived lines including substitution and translocation lines have been identified using this method [41–43]. In addition, structural changes in wheat chromosomes can be detected by FISH. For example, FISH was used to confirm a reciprocal translocation of the 5BL-7BS chromosome in a wheat variety Humai15 and a reciprocal translocation of the 4BL-6AL chromosome in a wheat variety Mingxian 169 [44]. In this study, however, FISH did not detect 5DL-5NsL.5NsS chromosome, therefore, 5NsL**·**5NsS might undergo another translocation within wheat chromosome, which is confirmed by FISH identification of two translocations, 3DS-5NsL**·**5NsS and 5DL-3DS**·**3DL in TR77. A previous FISH karyotype study on line 7182 did not find translocation between wheat chromosomes 3D and 5D [29]. Thus, the first translocation might have occurred between the wheat 5D and *P. huashanica* 5Ns chromosomes to yield chromosome 5DL-5NsL**·**5NsS as transition, and then a reciprocal translocation occurred between the wheat 3D and 5DL-5NsL**·**.5NsS chromosomes that produced 3DS-5NsL**·**5NsS and 5DL-3DS**·**3DL in TR77. During the two translocation events, TR77 lost its short arm and partial long arm of wheat 5D chromosome and acquired short arm (5NsS) and partial long arm (5NsL) of *P. huashanica* chromosome 5. Therefore, TR77 is a wheat–*P. huashanica* translocation line with dual translocations of 3DS-5NsL**·**5NsS and 5DL-3DS**·**3DL. The 5NsL**·**5NsS segment was combined with the wheat 3D chromosome via inter-chromosomal recombination. The fifth group of chromosomes in wheat carries the *Ph1* gene that controls the cross ability among species [45]. A previous study found that the wheat 5BL chromosome contained a subtelomeric insertion from wheat chromosome 3A [46]. Also a wheat line carrying T5BS.7BS, T5BL.7BL and T5AS.3BS chromosomes was identified using FISH technology [47]. Those studies support the possible reciprocal translocations between wheat chromosomes 5D and 3D occurred in this study.

Powdery mildew resistance genes from wild relatives of wheat are usually durable with broad-spectrum of resistance [48]. *Pm21* was transferred from *Haynaldia villosa* 6VS to wheat and a highly powdery mildew resistant 6VS/6AL translocation line is still effective and widely used in production [27]. The current study showed that TR77 was near immune to powdery mildew in both seedling and adult ages, although its two wheat parents exhibited susceptible symptoms, indicating that powdery mildew resistance trait in TR77 was from *P. huashanica*. So, the identification of TR77 filled a gap where no wheat–*P. huashanica* derived lines had excellent powdery mildew resistance before. Agronomic traits are important criteria for assessing the value of a line carrying alien chromosome segments. TR77 had larger spikes and more kernels per spike than its wheat parents, thereby has potential to increase yield. Crossing TR77 with locally adapted varieties can transfer these desired agronomic traits to new wheat cultivars. Line TR77 exhibited many excellent characteristics, which meant these excellent genes most possible introduced from *P. huashanica* because of chromosomal exchange and recombination between wheat and *P. huashanica.* The replaced segment were entire short arm and partial long arm of the chromosome close to centromere which was generally considered to contain few expressible genes. Therefore, the gene(s) that control awn length may be located in chromosome 5DS and raise powdery mildew resistance and spikes development may be located in chromosome 5NsS.

## Conclusion

In this study, TR77 was identified as a new wheat–*P. huashanica* T3DS-5NsL**·**5NsS and T5DL-3DS**·**3DL dual translocation line with larger spikes, more kernels per spike, and better powdery mildew resistance than their wheat parents. Molecular, cytogenetic and phenotypic analyses on TR77 determined its chromosomal composition, translocation position and inheritance stability as well as its superior agronomic performance. TR77 can be an important germplasm for improving powdery mildew resistance and yields in wheat breeding, and for dual translocation research.

## Methods

### Plant materials

The plant materials include one line of *Psathyrostachys huashanica* Keng (2*n* = 14, NsNs) from Huashan Mountains, Shaanxi province, China, one common wheat (*Triticum aestivum* L.) line 7182 (2*n* = 42, AABBDD), one durum (*Triticum durum*) line Trs-372 (2*n* = 28, AABB), and the wheat–*P. huashanica* translocation line TR77 obtained from the F_7_ progeny of the cross between line Trs-372 and a wheat–*P. huashanica* derived line H8911(2*n* = 49, AABBDDNs)*.* The heptaploid line H8911 was created by the wide cross between *P. huashanica* and line 7182 via artificial embryo rescue and backcross [8]. Common wheat varieties Huixianhong, Mianyang 11, and Chinese Spring were used as controls for assessing disease resistance.

The wheat wild related material *P. huashanica* was identified and collected by Shuyang Chen and Langran Xu who were the first researchers to do hybridization between common wheat and *P. huashanica* in China in 1980s [8]. The wheat materials were collected from The National Wheat Improvement Center of China and the wheat–*P. huashanica* derived lines were developed by our lab. All materials were deposited and planted in College of Agronomy, Northwest A&F University, China. The collection and treatment of materials for this experiment complied Wild Plants Protection Regulation of China. Extraction and purification of genomic DNA from leaf tissues used the standard Cetyltrimethylammonium Ammonium Bromide method [49].

### Cytological analysis

The appropriate stages for sampling were length of roots to 1.5 cm and young panicles to 5 cm [50]. The cut roots were immediately soaked in ice-water mixture for 12–20 h before transferring to Carnoy’s fixative fluid I for 24 h, and finally stored at − 20 °C. Young panicles at appropriate stage were treated with Carnoy’s fixative fluid II for 24 h and then stored under refrigeration. The root apical meristem was digested in 2% cellulase plus 1% pectinase at 37 °C for more than 50 min before scattering in acetic acid. The chromosomes in root tip cell were observed under a microscope at 400 times magnification (OLYMPUS BH2, Japan). Anthers were stained with 1% acetocarmine and gently broke for cytological observations. The slides with good split phases were dried and marked for the following experiments. Microscopic observations were the first step in order to identify the chromosomal number and behavior in TR77. In this process, each plant was numbered to ensure root and spike from the same plants.

### DNA marker analysis

A total of 384 pairs of SSR primers [51, 52] were selected from 21 wheat chromosomes to determine the translocated wheat chromosomes in TR77. In addition, to identify the homoeologous group of the introduced alien chromosomes in TR77, 87 pairs of EST-STS markers (https://wheat.pw.usda.gov/SNP/new/pcr_primers.shtml) were selected from seven wheat homoeologous groups that had homoeology with corresponding chromosomes of *P. huashanica*. PCR assays were conducted following Doğrar et al. [53] and an ABI PRISM 3730 DNA Analyzer (Applied Biosystems, USA) was adopted for separation of the PCR products. The data of SSR markers were scored by GeneMarker V1.97 (Soft Genetics LLC, USA).

### In-situ hybridization

For the GISH analysis, Mixed the *P. huashanica* genomic DNA and DiG-Nick Translation Mix (Roche, Germany) in a ratio of two to one at 15 °C for 1.5 h as labeling probes following Wetzel et al. [54] and Zhao et al. [55]. The addition of Anti-Digoxigenin–Fluorescein mix (Roche, Germany) made the probes visualize after hybridization. The wheat chromosomes were dyed using Vectashield H1300 (Vector Laboratories, USA). In the FISH analysis, the match of Oligo-pSc119.2 (6-FAM-5′CCGTTTTGTG GACTATTACT CACCGCTTTG GGGTCCCATA GCTAT) and Oligo-pTa535–1 (Tamra-5′AAAAACTTGA CGCACGTCAC GTACAAATTG GACAAACTCT TTCGGAGTAT CAGGGTTC) were used to give insight into the chromosomal composition of TR77. After hybridizing with wheat chromosomes, the standard karyotype of the two Oligo-probes could be seen [28, 39]. FISH experiment was conducted as described by Patokar et al. [56] and Lang et al. [57]. The signals were observed and captured using OLYMPUS BX60 fluorescence microscope with a color camera (Penguin, Japan) at 400 times magnification.

### Characterization of wheat-*P. huashanica* translocation using wheat 15 K SNP arrays

High-quality genomic DNA of TR77 and its parents was used to hybrid with Illumina wheat 15 K SNP arrays for loci difference scanning in China Golden Marker Biotechnology Company (Beijing, China). The array contained 13,199 SNP loci, which were dispersed in all 21 wheat chromosomes. The total valid number of SNP loci divided by the loci number that had the same genotype in a chromosome between materials was calculated as the percentage of the same markers in each chromosome. Data analysis used SigmaPlot V12.5 (SYSTAT software, USA) and chromosomal map was drew using MapChart V2.32 (Wageningen University & Research, The Netherlands).

### Characterization of agronomic traits and powdery mildew resistance

*P. huashanica*, TR77 and its wheat parents were evaluated in the field for seven agronomic traits, i.e. spike length, plant height, spikelet number, tiller number, kernel number, thousand-kernel weight and self-fertility. The mean values over five plants were collected in two successive years as repeats to ensure data accuracy.

The powdery mildew resistance was evaluated at the seedling stage in a growth chamber. The powdery mildew *Bgt* race E09 was used for inoculation with wheat variety Huixianhong as a susceptible control. The pathogen spores were dusted onto wheat leaves at two-leaf stage and incubated at 22 °C and 70% humidity for 15 d. the adult plant powdery mildew resistance was evaluated in a field in Sichuan province, China using Mianyang 11 as the susceptible control. The field usually had a high incidence of powdery mildew and thus tested plants were infected naturally. The reactions to powdery mildew were assessed following Sheng [58] and An [59]. In brief, wheat responses to infection were scored using infection type (IT) in a 0–4 IT scale at the seedling stage, in which IT = 0, 0;, 1, 2, 3 and 4 denoting immune, nearly immune, highly resistant, moderately resistant, moderately susceptible and susceptible respectively. In the adult stage, the plant responses to infection were recorded as the percentage of the powdery mildew spores covered the total area of the leaves at the same position on each plant [60].

## Supplementary information

**Additional file 1.** Figure S1 TR77-Molecular markers uncropped images.

**Additional file 2.** Table S1 TR77-SSR Analysis.

**Additional file 3.** Table S2 TR77-15K SNP array.

## Data Availability

All related plant materials are available and comply Wild Plants Protection Regulation of China. The datasets supporting the conclusions of this article are included within the article and its supplementary files.

## Abbreviations

EST-STS: Expressed sequence tag-sequence tagged site
FISH: Fluorescence in situ hybridization
GISH: Genomic in situ hybridization
IT: Immunity type
*P. huashanica*: *Psathyrostachys huashanica* Keng
PMC: Pollen mother cell
SCAR: Sequence characterized amplified region
SNP: Single nucleotide polymorphism
SSR: Simple sequence repeat
RTC: Root tip cell
