# Supplementary material for: Molecular cytogenetic characterization of a novel wheat–Psathyrostachys huashanica Keng T3DS-5NsL•5NsS and T5DL-3DS•3DL dual translocation line with powdery mildew resistance
Source: BMC Plant Biol. 2020 Apr 15;20:163. doi: 10.1186/s12870-020-02366-8 (PMC7161236; doi:10.1186/s12870-020-02366-8)

STS markers analysis

Lane 1: DNA marker ; lane 2: 7182; lane 3: Trs-372; lane 4: TR77 ; lane 5: *P. huashanica.*

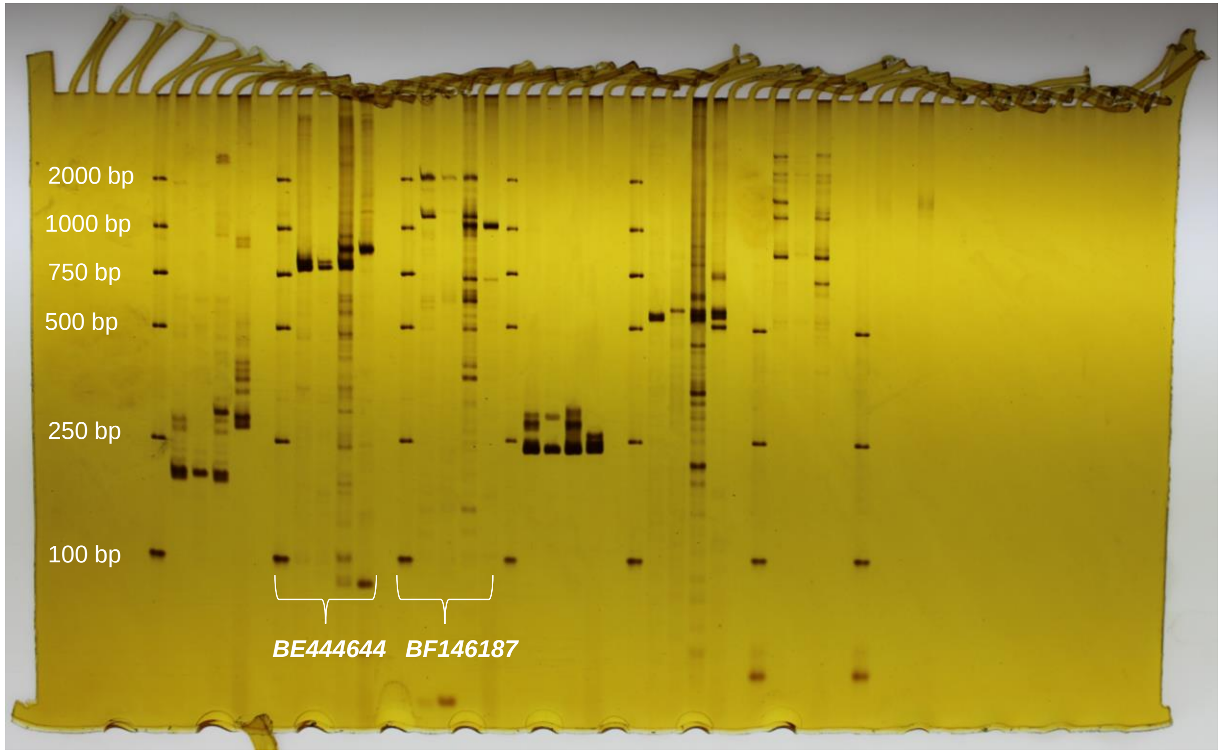

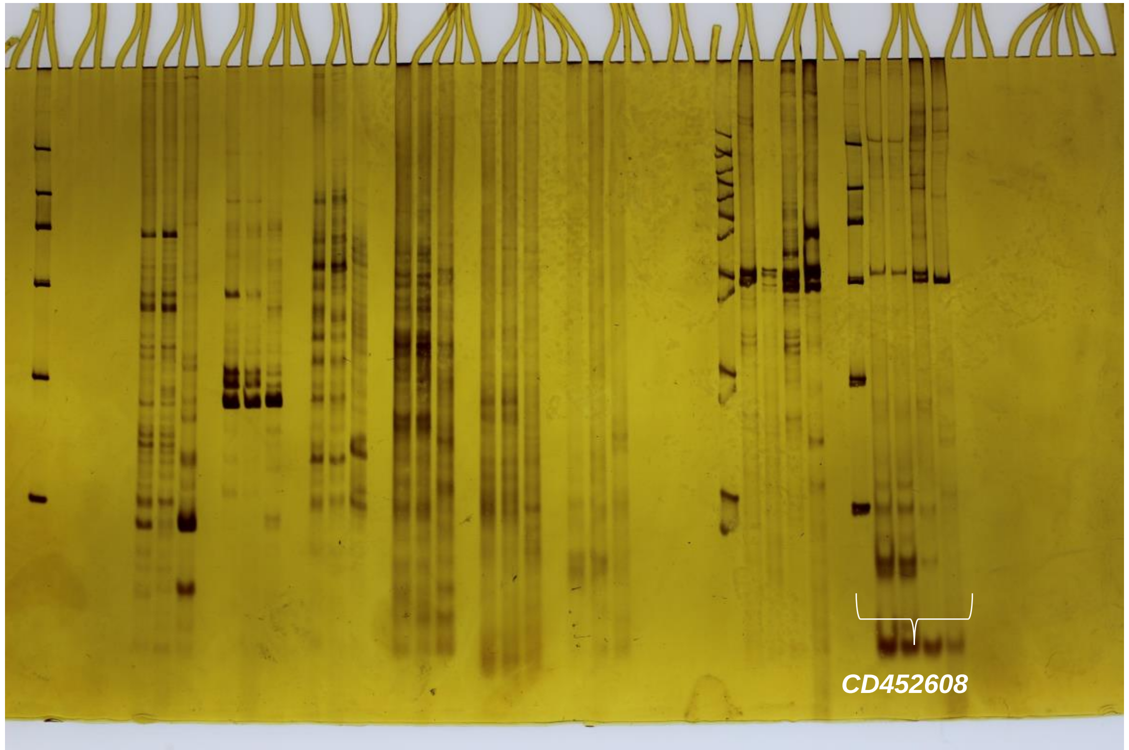

SSR markers analysis

Lane 1: DNA marker ; lane 2: 7182; lane 3: Trs-372; lane 4: TR77.

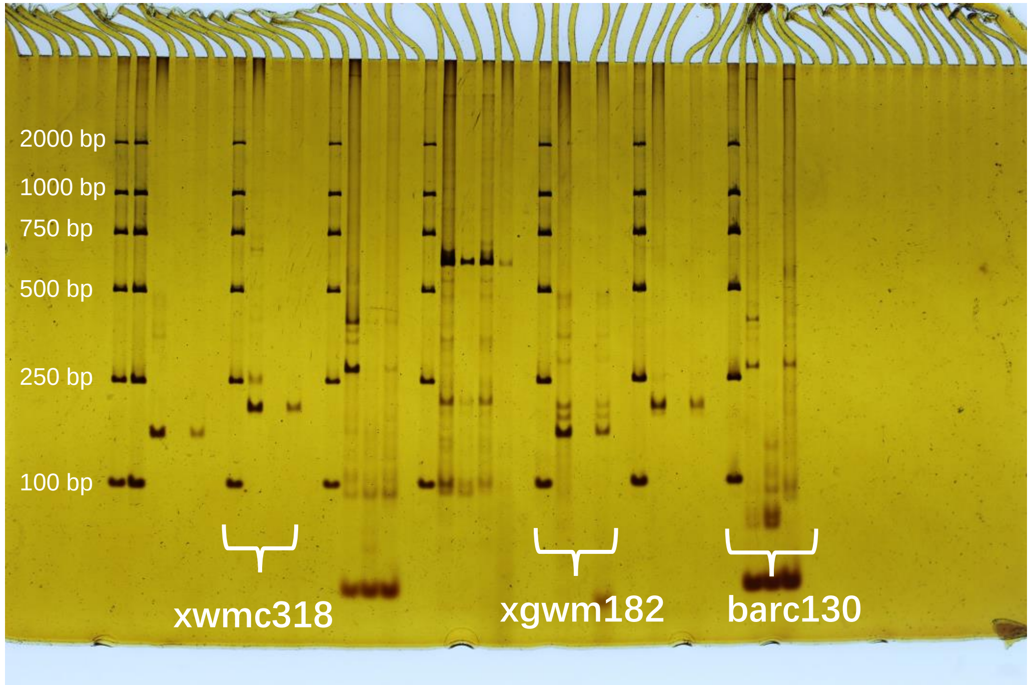

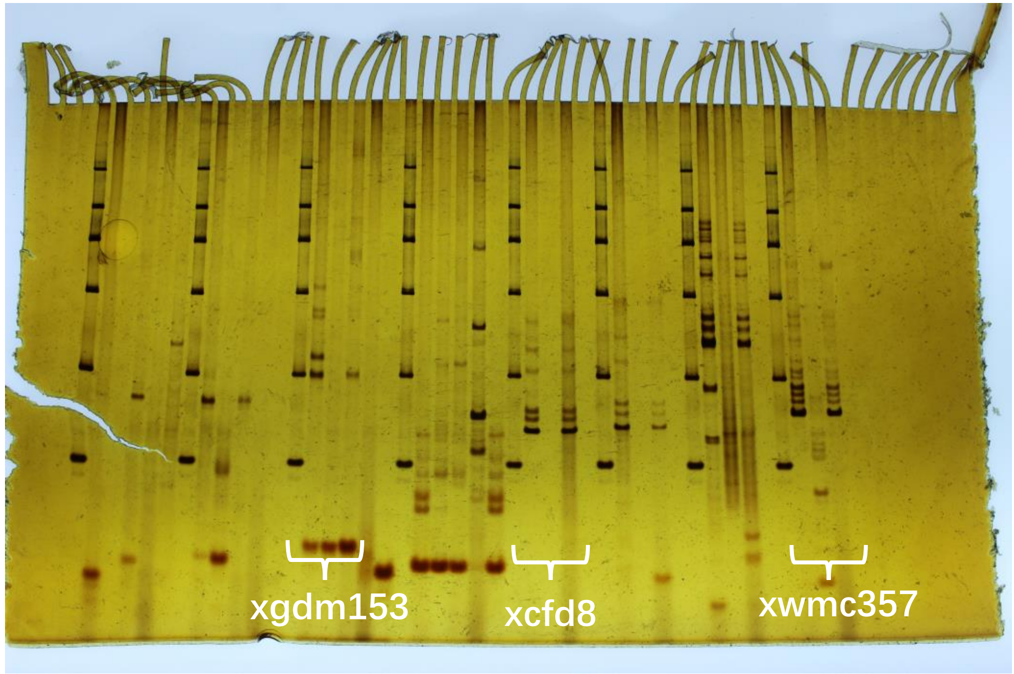

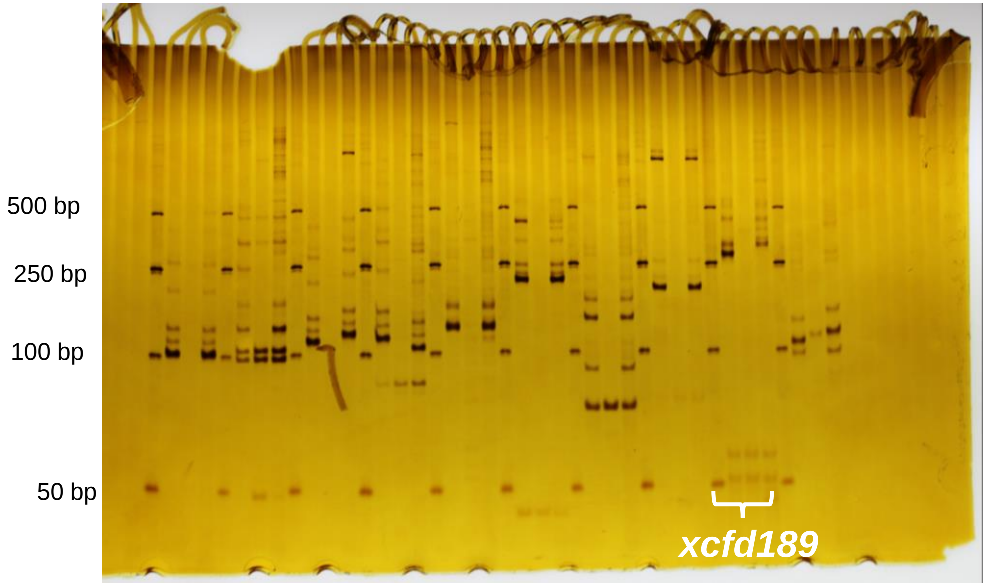

Supplement: Supplementary file 1 — Additional file 1. Figure S1 TR77-Molecular markers uncropped images. [file 12870_2020_2366_MOESM1_ESM.pdf]
